# 液相色谱-离子色谱联用体系同时测定环境基质中的6种硝基芳烃类化合物和3种阴离子

**DOI:** 10.3724/SP.J.1123.2023.10027

**Published:** 2024-01-08

**Authors:** Jiacheng LEI, Huangrong ZHENG, Lu LIU, Weixia LI

**Affiliations:** 1.中国计量大学标准化学院, 浙江 杭州 310018; 1. College of Standardization, China Jiliang University, Hangzhou 310018, China; 2.中国标准化协会, 北京 100037; 2. China Association for Standardization, Beijing 100037, China

**Keywords:** 离子色谱, 高效液相色谱, 硝基芳烃类化合物, 阴离子, 土壤, 环境水, ion chromatography (IC), high performance liquid chromatography (HPLC), nitroaromatic compounds, anions, soil, environmental water

## Abstract

同时测定环境土壤和水基质中的硝基芳烃类化合物和阴离子对于选择合适的硝基芳烃类化合物降解方法和实现地表水水质监测具有重要意义。本文通过两个六通阀和一根富集柱将高效液相色谱和离子色谱连接,搭建了色谱联用体系。系统操作可分为4个阶段:(A)样品加载到定量环;(B)分离硝基芳烃类化合物和阴离子;(C)阴离子在AG20柱中富集;(D)高效液相色谱和离子色谱分别测定硝基芳烃类化合物和阴离子。通过将液相色谱柱直接连接电导率检测器,确定阴离子流出液相色谱柱的时间,优化六通阀的切换时间,保证方法的准确性。在最优条件下,阴离子的检出限为0.005~0.020 mg/L,硝基芳烃类化合物的检出限为0.043~0.150 mg/L。3组不同加标浓度样品的平均回收率为88.20%~105.38%,相对标准偏差为2.0%~11.5%。该方法被用于检测5份地表水样品和5份土壤样品,硝基芳烃类化合物均未检出,3种阴离子在地表水样品中的检出量为0.41~55.3 mg/L,在土壤样品中的检出量为0.56~30.2 mg/kg。经过方法学验证和实际样品检测,证明该色谱联用体系检测方法自动化程度高,操作简单,重复性好,准确度高,具有广泛的适用性和较高的灵敏度,适用于环境基质中硝基芳烃类有机物和亚硝酸根离子等阴离子含量的快速测定。

## Abstract (en)

Nitroaromatic compounds are used extensively in various fields such as dyes, pesticides, spices, pharmaceuticals, and explosives. However, the residual raw materials of these compounds accumulate in the environment and pose serious risks to human health. Chronic exposure to low concentrations of nitroaromatic compounds can cause anemia, cancer, and organ damage. Currently, Fenton oxidation and natural bioremediation are the processes most often used to eliminate nitroaromatic compounds from environmental water and soil. According to previous research, the presence of inorganic anions such as chloride, nitrite, and nitrate ions in the environmental matrix exerts an inhibitory effect on the biodegradation of nitroaromatic compounds. Furthermore, high nitrate levels in drinking water can lead to the production of nitrosamine carcinogens, which affect ecological safety and human health, in water bodies. Thus, the simultaneous determination of nitroaromatic compounds and chloride, nitrite, and nitrate ions in environmental soil and water matrices is critical for selecting appropriate nitroaromatic compound degradation methods and monitoring surface water quality. Traditional detection methods require two sample pretreatment steps and two instrumental analytical techniques to determine nitroaromatic compounds and inorganic anions in environmental matrices; moreover, these methods are time consuming, labor intensive, and error prone. Therefore, in this study, a method that combines high performance liquid chromatography (HPLC) and ion chromatography (IC) was developed to simultaneously detect nitroaromatic compounds and anions in environmental matrices. In this method, sample enrichment was achieved through bulk injection and enrichment column collection, which greatly simplified the pretreatment process. The HPLC instrument was connected to the IC instrument using two six-way valves and an enrichment column. The system operation can be divided into four stages: (A) sample loading to the quantitative ring, (B) separation of nitroaromatic compounds and anions, (C) enrichment of anions in an AG20 column, and (D) simultaneous determination of nitroaromatic compounds and anions by HPLC and IC, respectively. The time of the anions flowing out of the C18 column was determined by directly connecting the C18 column to a conductivity detector. Based on the retention times of the anions, the switching time of the six-way valve was optimized to ensure that the anions completely entered the IC column, thereby ensuring the accuracy of the method. During the chromatographic analysis stage, nitroaromatic compounds were separated and analyzed by HPLC system with a mobile phase composed of potassium phosphate buffer (pH 7.0) and acetonitrile (60∶40, v/v) at a flow rate of 1.0 mL/min; in the IC system, the anions were separated and analyzed using a 20 mmol/L sodium hydroxide aqueous solution as the mobile phase under a suppression current of 50 mA. Both anions and nitroaromatic compounds exhibited strong linear correlations within certain concentration ranges, with correlation coefficients greater than 0.993. The recoveries of the nitroaromatic compounds and anions ranged from 88.20% to 105.38% at three spiked levels, with relative standard deviations ranging from 2.0% to 11.5%. The contents of six nitroaromatic compounds and three anions in five surface water and five soil samples were determined using the developed method. Although no nitroaromatic compounds were detected in these samples, the three anions were detected at contents ranging from 0.41 to 55.3 mg/L in surface water samples, and 0.56 to 30.2 mg/kg in soil samples. Methodological validation and actual sample detection demonstrated that the proposed method has a high degree of automation, simple operation, good repeatability, high accuracy, wide applicability, and high sensitivity. Thus, this method is suitable for the rapid determination of chloride, nitrite, nitrate ions and nitroaromatic compounds in soil and water and can be extended to the simultaneous determination of inorganic ions and organic matters in other samples.

硝基芳烃类化合物(NACs)是具有一个或多个硝基基团的芳香族化合物,包括硝基苯(NB)、2,4-二硝基甲苯(DNT)、2,4,6-三硝基苯酚(TNP)等,作为染料、农药、香料、药品、炸药等产品的原料或中间体被广泛应用^[1⇓-3]^。各种军事活动中残留的爆炸物会随着雨水冲刷进入环境水体,造成污染。此外,生活污水排放、药厂废水排放、石油开采等行为同样会造成硝基芳烃类化合物环境污染。环境中残留的硝基芳烃类化合物对人体健康有巨大威胁,长期暴露在低浓度硝基芳烃类化合物污染的环境中会造成人体器官受损、贫血和致癌等疾病^[4]^。我国的强制标准《地表水环境质量标准》(GB 3838-2002)规定集中式生活饮用水地表水源地中硝基苯含量必须低于0.017 mg/L^[5]^。实时监测并有效消除环境水体中的硝基芳烃类化合物对实现用水安全意义重大。

Fenton氧化法、自然生物修复工艺是常见的环境水体中硝基芳烃类化合物消除办法^[6,7]^。Fenton氧化法对环境水体中硝基芳烃类化合物的消除效果好,但操作麻烦,费用较高。自然生物修复工艺利用微生物对污染土壤中的硝基芳烃类化合物进行降解,是目前常用的硝基芳烃类化合物的绿色消除方法。但是研究表明环境基质中的氯离子、亚硝酸根离子、硝酸根离子等无机阴离子的存在对硝基芳烃类化合物的生物降解具有一定的抑制作用^[8,9]^。另外,硝酸盐如果在饮用水中含量过高会诱发水体产生亚硝胺类致癌物质,GB 3838-2002规定水体中硝酸盐类离子的含量应低于10 mg/L^[10]^。因此,同时测定环境土壤和水体基质中的硝基芳烃类化合物、氯离子、亚硝酸根离子和硝酸根离子对选择合适的硝基芳烃类化合物降解方法和实现地表水质量监测具有重要意义。

环境基质中的硝基芳烃类化合物通常通过气相色谱、高效液相色谱(HPLC)、荧光探针、电化学等方法进行检测^[11⇓⇓⇓-15]^,而其中的无机阴离子则采用离子色谱(IC)进行检测^[16,17]^。传统的检测方法需要进行两次样品前处理、两次仪器分析才能实现环境基质中硝基芳烃类化合物和无机阴离子的测定,耗时费力,且容易引入误差。因此,本论文通过串联HPLC和IC构建二维色谱体系,实现了环境基质中有机物和阴离子的同时测定。该方法无需复杂的样品前处理,通过大体积进样-富集柱收集的方法实现目标化合物的富集,具有简单高效、检出限低、重复性高等优点,在环境监测领域具有一定的应用潜力。

## 1 实验部分

### 1.1 仪器

本实验所搭建的联用系统包含一个HPLC系统和一个IC系统。通过两个六通阀和一根富集柱联接两台仪器,具体如图1所示。UltiMate 3000 UHPLC超高效液相色谱(美国Thermo Fisher公司),配有四元梯度泵、自动进样器、柱温箱和紫外检测器。ICS-2100离子色谱仪(美国Thermo Fisher公司),配备淋洗液发生器、六通阀、柱温箱、抑制器和DS6电导检测器。仪器控制和数据采集采用Chromeleon 6.8色谱工作站。

**图1:**
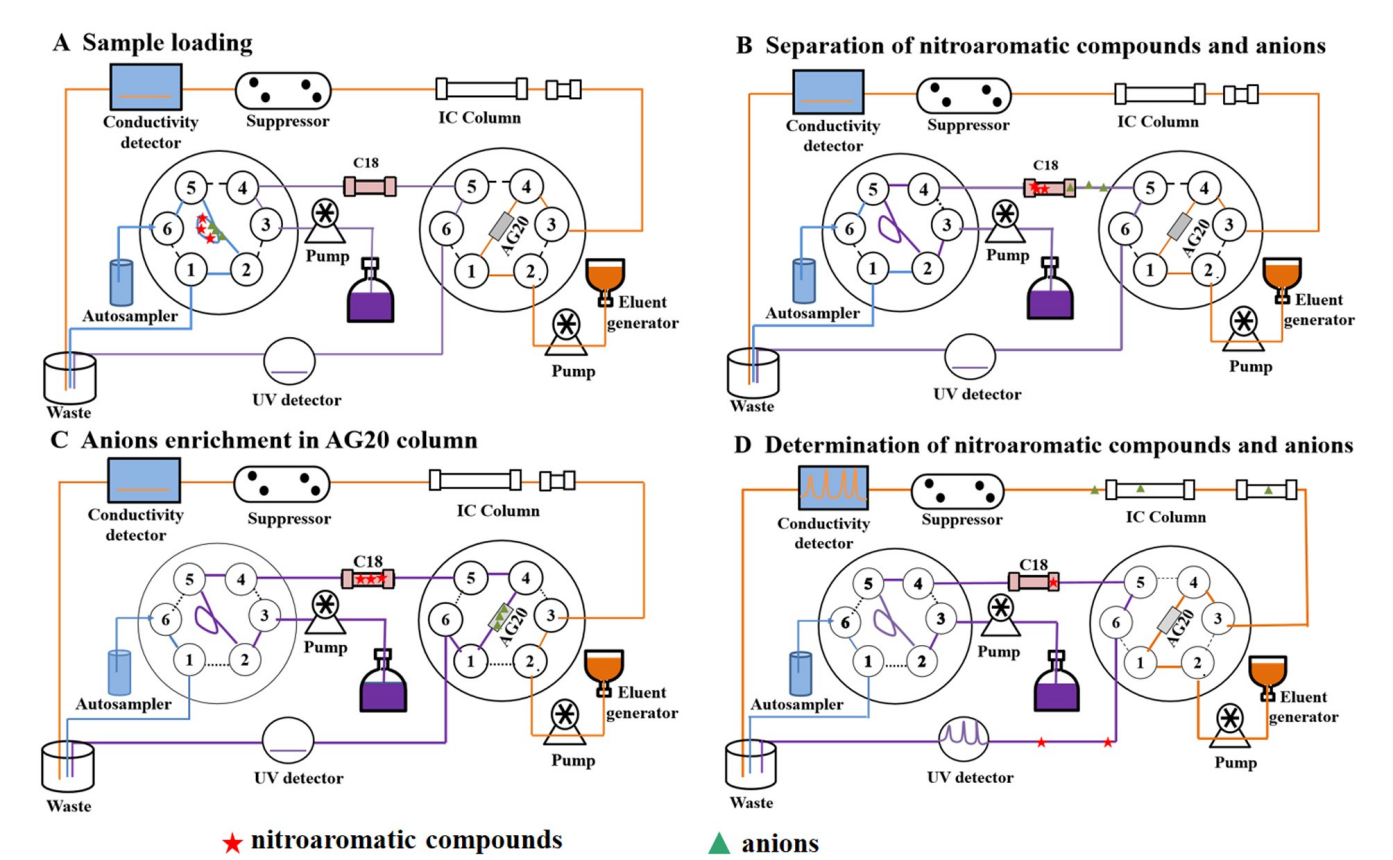
HPLC-IC联用体系4个阶段的液体流路示意图

### 1.2 试剂和样品

HPLC级甲醇、1,3,5,7-四硝基-1,3,5,7-四氮杂环辛烷(HMX)、环三亚甲基三硝胺(RDX)、1,3,5-三硝基甲苯(TNB)、1,3-二硝基苯、2,4,6-三硝基甲苯(TNT)、2,4-二硝基甲苯均购自上海阿拉丁化工有限公司。氯化钠、亚硝酸钠和硝酸钠购自成都科龙化学试剂厂。实验用水均为二次去离子水。

标准储备液:将氯化钠、亚硝酸钠和硝酸钠使用前置于马弗炉中于105 ℃下干燥3 h除去水分。准确称取氯化钠、亚硝酸钠、硝酸钠和各硝基芳烃类化合物,用超纯水定容至100 mL,得到含量均为1000 mg/L的混合标准储备液,混匀,超声脱气,储存于4 ℃冰箱内。实验所用的标准工作溶液均由储备液根据需要用水稀释而成。

### 1.3 样品前处理条件

收集5份土壤样品,烘干,并用研钵碾碎,以孔径为20目的筛进行筛选^[18]^。取过筛的土壤样品10.0 g置于烧杯中,加入30.0 mL甲醇/水(50∶50, v/v)混合溶液,超声浸提20 min后,以4000 r/min的速度离心10 min,取上清液2.0 mL,过0.22 μm滤膜后进样分析。收集5份环境水样,反复过0.22 μm滤膜3次后进样分析。

### 1.4 仪器分析

将HPLC仪和IC仪按图1A所示方法进行连接,此过程中应尽量缩短流路以减少死体积。液相色谱柱为Acclaim Explosives E1(250 mm×4.6 mm, 5 μm,美国Thermo Fisher公司),柱温设定为32 ℃;流动相组成为磷酸钾缓冲液(pH 7.0)-乙腈(60∶40, v/v),等度淋洗,流速1.0 mL/min;进样量500 μL;紫外检测波长为223 nm。离子色谱柱为IonPac AG11-HC预处理柱(50 mm×4 mm, 5 μm,美国Thermo Fisher公司)和IonPac AS11-HC柱(250 mm×4 mm, 5 μm,美国Thermo Fisher公司),柱温箱温度为40 ℃;等度淋洗,流动相为20 mmol/LNaOH溶液,抑制器的电流设置为50 mA。在此联用系统中,AG20-HC预处理柱(50 mm×4 mm, 5 μm,美国Thermo Fisher公司)作为富集柱,被安装在六通阀上。

图1为HPLC-IC联用体系柱切换的示意图,表1为两个六通阀的状态。整个联用系统的具体操作步骤如下:(A)样品装载到定量环:将待测样品注入定量环,多余样品进入废液缸。(B)分离有机物和无机阴离子:使用HPLC流动相冲洗定量环,将定量环中的待测样品注入C18柱中,进行第一次分离;硝基芳烃类化合物在C18柱中保留能力强,保留在C18色谱柱中;阴离子在C18柱中保留能力弱,随流动相流出。(C)AG20柱富集阴离子:流出的阴离子进入AG20富集柱富集。(D)HPLC和IC分别测定6种硝基芳烃类化合物和3种阴离子:硝基芳烃类化合物在C18色谱柱中分离,然后进入紫外检测器定量分析;IC流动相冲洗AG20富集柱,阴离子从AG20富集柱中流出后在IC柱中分离,然后通过抑制器进入电导检测器中定量分析。

**表1:** HPLC-IC联用体系两个六通阀的切换状态

Stage in Fig. 1	Operation process	Time/min	Left six-way valve	Right six-way valve
A	sample loading to quantitative ring	-	loading	injection
B	separation of nitroaromatic compounds and anions in C18 column	0-1.5	injection	injection
C	anions enrichment in AG20 column	1.5-3.0	injection	loading
D	determination of nitroaromatic compounds and anions by HPLC and	3.0-20	injection	injection
	IC, respectively			

## 2 结果与讨论

### 2.1 柱切换时间优化

为了避免阴离子流失,对六通阀的切换时间进行优化。如图2A所示,将C18柱直接与DS6电导检测器相连,对阴离子的收集行为进行观察。以10 mg/L的阴离子混合标准溶液为样品,进样后,离子经过C18色谱柱直接流入DS6电导检测器,得到图2B所示的非抑制电导IC图。1.5 min时,DS6电导检测器开始检测出离子,可认为1.5 min时,离子开始从C18柱中流出,因此HPLC-IC联用系统由图1B转变至图1C的时间节点定为1.5 min。

**图2:**
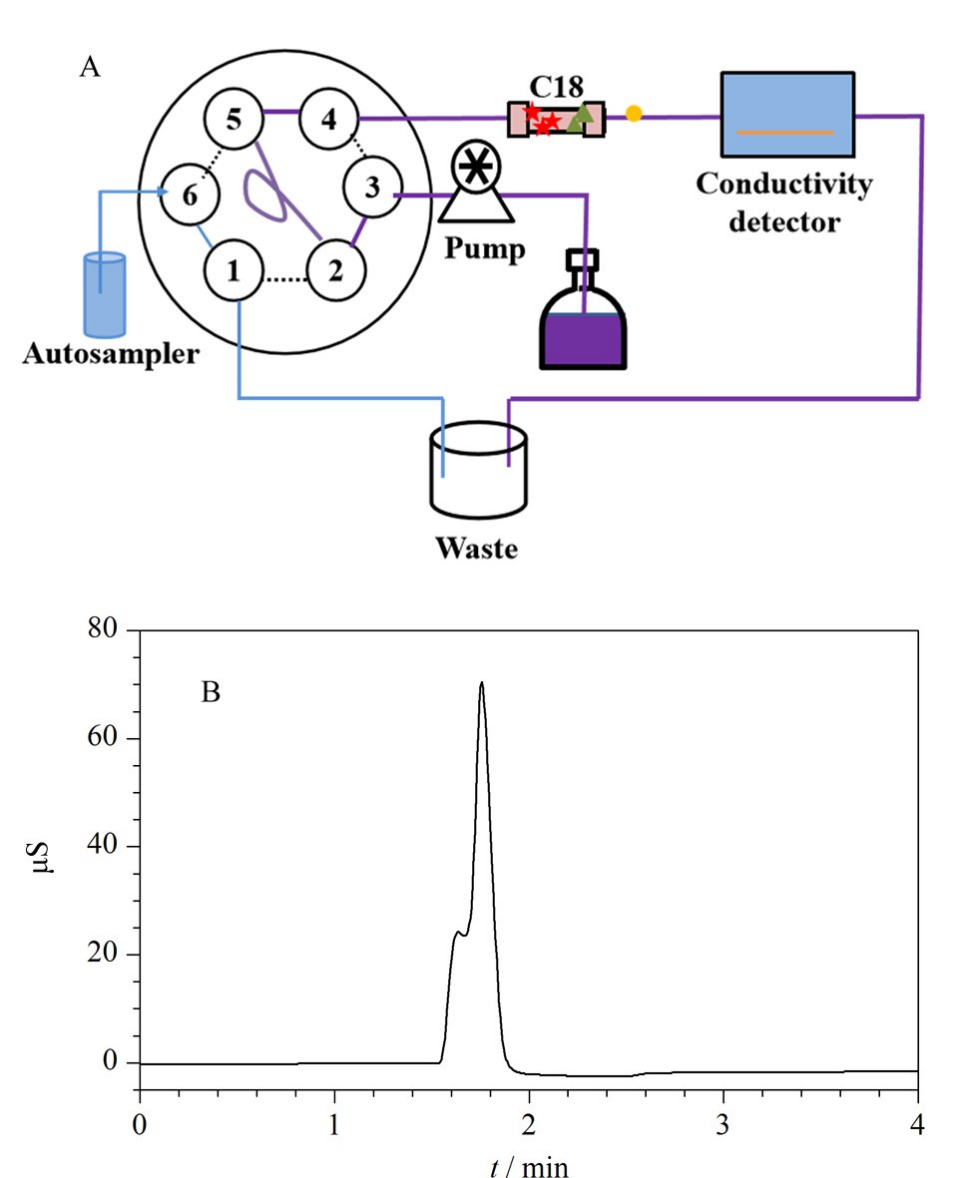
(A)柱切换时间优化的装置示意图和(B)阴离子的非抑制电导色谱图

为提高离子的回收率,对阴离子进行完全富集,进一步优化两个六通阀的切换时间。选择不同富集时间,即选择在不同时间将联用系统从图1C状态切换到图1D状态。通过对比不同切换时间下获得的各离子峰面积可知,当切换时间为3.0 min时,氯离子、亚硝酸根离子和硝酸根离子的峰面积达到峰值(图3)。当时间大于3.0 min后,部分离子从富集柱中流失,离子的富集效果开始变差。因此系统从图1C状态切换到图1D状态的时间应设置为3.0 min。

**图3:**
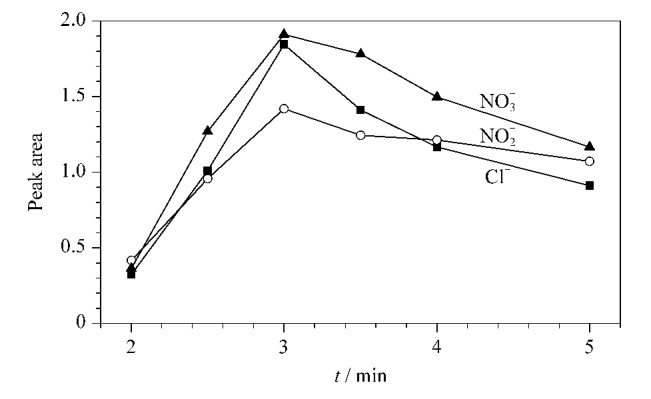
不同柱切换时间下的阴离子峰面积

### 2.2 方法学验证

#### 2.2.1 线性关系和检出限

用空白土壤为基质制备加标水平为0.01~50 mg/L的加标样品,在最优色谱分析条件下进行测定,每个水平平行测定5次,以待测物峰面积对质量浓度绘制工作曲线,得到方法的线性范围、线性回归方程和相关系数(*r*^2^),结果见表2。*r*^2^均大于0.99,满足测定要求。5次重复试验结果表明,保留时间的相对标准偏差(RSD)≤0.28%,峰面积的RSD≤5.0%,表明该方法具有较高的重复性。

**表2:** 线性参数、检出限、定量限和重复性

Analyte	Linear range/ (mg/L)	Correlation coefficient (r^2^)	LOD/ (mg/L)	LOQ/ (mg/L)	RSDs/%(n=5)	
					Retention time	Peak area
HMX	0.49-50	0.9973	0.150	0.49	0.17	4.2
RDX	0.35-50	0.9999	0.112	0.35	0.14	4.2
TNB	0.31-10	0.9931	0.089	0.31	0.15	5.0
1,3-Dinitrobenzene	0.29-50	0.9996	0.085	0.29	0.14	4.2
TNT	0.18-10	0.9997	0.057	0.18	0.13	4.5
2,4-Dinitrotoluene	0.13-50	0.9978	0.043	0.13	0.04	3.6
Cl^-^	0.016-25	0.9991	0.005	0.016	0.28	5.0
N	0.049-40	0.9991	0.016	0.049	0.28	3.5
N	0.059-40	0.9993	0.020	0.059	0.25	1.6

HMX: 1,3,5,7-tetranitro-1,3,5,7-tetranitrocyclohexane; RDX: cyclotrimethylene trinitroamine; TNB: 1,3,5-trinitrotoluene; TNT: 2,4,6-trinitrotoluene.

在最优实验条件下,以信噪比(*S/N*)为3作为检出限(LOD),以*S/N*为10作为定量限(LOQ),对实际样品逐级稀释测定。阴离子的LOD为0.005~0.020 mg/L,硝基芳烃类化合物的LOD为0.043~0.150 mg/L。现行地表饮用水规范的国标方法中,硝酸盐含量不得高于10 mg/L,二硝基苯和TNT含量不得高于0.5 mg/L,该方法能够满足地表水样品中硝基芳烃类化合物和硝酸盐的检测需求。

#### 2.2.2 方法的回收率和精密度

选择阴性土壤和环境水样品,分别添加低、中、高3个不同水平的混合标准溶液。按1.3节进行前处理,每个水平重复测定6次,计算加标回收率和RSD,结果见表3。三组不同加标水平样品的平均回收率为88.20%~105.38%,RSD为2.0%~11.5%,表明本方法具有较高的准确度。

**表3:** 6种硝基芳烃类化合物和3种阴离子的加标回收率和相对标准偏差(*n*=6)

Analyte	Added/ (mg/L)	Recoveries/%			RSDs/%		Analyte	Added/ (mg/L)	Recoveries/%			RSDs/%			
		Soil	Water		Soil	Water			Soil	Water		Soil	Water		
HMX	0.22	98.27	90.83		2.7	6.7	2,4-Dinitrotoluene	0.56	102.11	91.34		7.3			7.4
	0.56	101.98	92.07		9.3	8.2		1.25	92.92	91.47		8.0			11.3
	1.45	100.05	103.62		5.0	9.5		2.78	88.20	89.95		8.1			5.1
RDX	0.28	92.63	96.63		7.1	2.1	Cl^-^	0.12	88.61	91.67		4.1			2.8
	0.56	105.38	93.96		11.5	5.3		1.25	91.73	88.43		11.0			2.4
	1.45	98.32	98.41		5.5	3.6		10.0	98.47	89.75		2.0			5.1
TNB	0.22	96.49	96.50		2.7	5.0	N	0.25	95.18	97.92		9.5			5.1
	0.45	98.25	89.89		9.9	4.5		2.50	100.88	89.37		3.1			3.7
	1.15	102.06	96.66		8.2	7.7		20.0	97.57	91.46		6.2			4.5
1,3-Dinitrobenzene	0.56	91.48	92.43		7.8	9.1	N	0.25	94.84	90.63		6.1			9.1
	1.25	100.41	91.57		8.5	6.6		2.50	97.64	94.06		4.3			2.1
	2.78	98.33	94.23		11.4	8.3		20.0	89.88	91.57		8.7			2.2
TNT	0.40	99.33	96.87		7.9	9.3									
	0.55	103.61	90.40		7.3	4.9									
	1.15	102.00	94.33		5.4	4.7									

### 2.3 实际样品测定

应用本研究建立的方法对5份土壤样品和5份环境水样中的硝基芳烃类化合物和阴离子进行检测。将检出的色谱峰保留时间与标准品色谱峰保留时间相比较,经确证,硝基芳烃类化合物均未检出,3种阴离子在地表水样品中的检出量为0.41~55.3 mg/L,在土壤样品中的检出量为0.56~30.2 mg/kg(见表4)。1号土壤样品的HPLC图和IC见图4。

**表4:** 5份地表水和5份土壤样品的检测结果

Analyte	Surface water samples/(mg/L)						Soil samples/(mg/kg)					
	1	2	3	4	5		1	2	3	4		5
Cl^-^	25.3	36.3	27.4	55.3	41.4		25.1	30.2	15.5	18.2		23.2
N	-	0.41	-	0.54	0.72		-	0.56	0.63	1.50		-
N	1.38	1.82	2.16	1.49	1.33		0.65	1.35	2.39	2.16		2.01

**图4:**
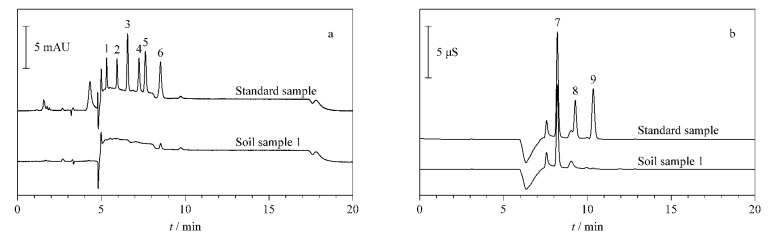
标准样品和1号土壤样品的(a) HPLC和(b) IC图

## 3 结论

本文通过两个六通阀和一根富集柱搭建了HPLC和IC的联用体系,并建立了土壤和环境水体中硝基芳烃类有机物、氯离子、亚硝酸根离子和硝酸根离子同时测定的检测方法。结果表明,该方法有良好的准确度、重复性和较高的灵敏度,能为选择合适的硝基芳烃类化合物降解方法和实现地表水水质监测提供重要的方法依据。
